# Communication and Social Relations: A Qualitative Study of Families’ Experience with Their Outpatient Pediatric Diabetes Visits

**DOI:** 10.3390/children9020245

**Published:** 2022-02-11

**Authors:** Louise Norman Jespersen, Jannet Svensson, Kasper Ascanius Pilgaard, Dan Grabowski

**Affiliations:** 1 Health Promotion Research, Copenhagen University Hospital—Steno Diabetes Center Copenhagen, DK-2730 Herlev, Denmark; jannet.svensson@regionh.dk (J.S.); kasper.ascanius.pilgaard.04@regionh.dk (K.A.P.); dan.grabowski@regionh.dk (D.G.); 2 Department of Pediatrics, Copenhagen University Hospital—Steno Diabetes Center Copenhagen, DK-2730 Herlev, Denmark

**Keywords:** diabetes, children, adolescents, family interviews, outpatient visits, communication, social relations

## Abstract

Clinical outpatient visits comprise a relatively small part of the lives of children with diabetes and their families, but there is evidence that these visits have a strong impact on the long-term management of diabetes. Because children with diabetes are looking at frequent hospital visits for the rest of their lives, it is important to explore their experiences to ensure visits meet their needs. This study aimed to investigate families’ experiences with outpatient visits at a pediatric diabetes clinic. Thirteen semi-structured family interviews were conducted. Systematic text condensation was used to analyze the data. With an analytical focus on communication and social relations, nine themes were identified: 1. Discrepancies in perception of diabetes tasks, 2. Talking about adult things, but the children listen, 3. The importance of spoken and written words, 4. Confusion about division of responsibilities, 5. Relief when someone eases the burden, 6. Courtesy when visiting the clinic, 7. Understanding of the family context, 8. Importance of continuous personal relations, and 9. Need for a facilitated peer network. The findings encourage reflection on how to improve communication and underline the importance of establishing a continuous and personal relation between families and health care professionals to improve families’ experience with pediatric outpatient visits.

## 1. Introduction

Each year, approximately 300 Danish children and adolescents are diagnosed with type 1 diabetes. This number is increasing by about 3% every year [1]. In Denmark, 90% of children with diabetes visit an outpatient clinic at least three times a year [2]. Although these clinical visits comprise a relatively small part of the families’ everyday lives, there is evidence that the experience of the visits influences the long-term management of diabetes [3]. Relationships built on trust are found to be important to the way in which children and adolescents with chronic illness experience communication with their health care professionals [4], and health care professionals believe that trusting relationships during clinical visits are important for younger children [5]. Moreover, children’s and adolescents’ involvement in discussions and decisions has been described as fundamental to the experience of communication. Examples of barriers to a well-functioning pediatric outpatient diabetes visit include: formal, rushed, and unsupportive communication [6], children experiencing that their suggestions about care are ignored [7], and not seeing the same doctor at each consultation [8]. Because children with diabetes are looking at frequent hospital visits for the rest of their lives, it is important to explore how they and their families experience the clinical visits and to use this knowledge to improve their visits.

When studying the lives of children with diabetes, inclusion of the parents is crucial. The extent of daily management and the responsibility of children with diabetes and their families are unlike the requirements associated with most other chronic illnesses, because diabetes necessitates a complex daily self-management treatment regime including blood glucose monitoring, insulin therapy, meal planning, and exercise [9]. Although health care professionals are important sources of expertise, children’s HbA1c levels are mainly determined by how they and their parents manage diabetes through daily self-management treatment [10]. When the child is young, this responsibility for diabetes management belongs solely to the parents, but as the child grows, the responsibility will gradually shift from the parent to the child. Hence, it is vital to consult and involve the parents, as their commitment and support are prerequisites for the well-being of the child [11,12]. Having to deal with illness management tasks in everyday life places a heavy burden on the children and their parents. This can lead to serious psychosocial problems, which may affect diabetes management and ultimately lead to poorer HbA1c in the children [13,14]. The International Society for Pediatric and Adolescent Diabetes (ISPAD) states that a general aim of outpatient visits should be to provide individualized care that best meets the family’s needs [15]. To accomplish this, in-depth knowledge about these needs and preferences is crucial.

Until now, children with diabetes (0–18 years) living in the Capital Region of Denmark have been treated at two different pediatric clinics. In both settings, the children attend the outpatient clinic 4–5 times a year and are followed by the same team of nurses and doctors, from diabetes onset to the transition to the adult diabetes department. The larger “Clinic A” treats about 600 children with diabetes, whereas the smaller “Clinic B” has approximately 240 children with diabetes enrolled. Yearly, a total of about 100 new patients are admitted to one of the clinics. In 2021, the two pediatric diabetes clinics were moved to new premises and merged into one new pediatric diabetes clinic at Steno Diabetes Center Copenhagen (SDCC). Differences in diabetes treatment practices across Danish pediatric clinics have previously been identified [2]. Researchers studying this concluded that the metabolic outcome of pediatric diabetes management in Denmark was unsatisfactory and suggested that studies be conducted exploring how metabolic regulation can be improved [2]. The intention of the new uniform practices at the new SDCC pediatric clinic is to adapt, adjust, or revise the existing practices from the two clinics to accommodate the new clinic. The restructuring of practices may provide an opportunity to incorporate into the new clinic experiences of positive, informal practices that are valued by children with diabetes and their families [16]. The aim of the present study was to explore families’ experience with outpatient visits at the pediatric clinics and to use the results to improve treatment at the new SDCC clinic.

## 2. Materials and Methods

We conducted a qualitative interview study specifically addressing experiences with pediatric outpatient visits.

### 2.1. Recruitment and Participants

The target group for the present study was children and young adolescents with type 1 diabetes and their parents. For recruitment, the researchers contacted nurses working at the two pediatric outpatient clinics, asking them to inform potential participants about the study. Families that were willing to learn more about the study and agreed that the nurse could share their contact details with the researchers were contacted by the first author by telephone in order to provide the families with more in-depth information about the study. Inclusion criteria were that the child was 3–15 years old and diagnosed with type 1 diabetes. It was further required that the child and at least one parent were interviewed together. In total, thirteen families were contacted, and all of them were enrolled in the study. The participating children were 3–15 years of age and of both genders. At the time of interviews, the duration of diabetes ranged from 0.5 to 9 years. The characteristics of participants are shown in Table 1. Data on socioeconomic status and HbA1c were not collected.

### 2.2. Data Collection

Data were collected via family interviews with 13 families in their own home. In one case (family number 10), the parents participated without their son, due to a misunderstanding. The family interviews took the form of participatory single-family workshops [17] with the following four elements:

(1) Introductory question about time of diagnosis;
(2) Drawing exercise (described below);
(3) Ranking exercise (described below);
(4) Supplementary questions.

The interviews were semi-structured, followed the same interview guide, and were conducted by the same researcher. Most interviews lasted about an hour (range 41–87 min)

The interviews were conducted within a social constructionist frame, which is a widely used approach in interview studies including several family members [18]. In line with this approach, we aimed to explore how children and their parents constructed and interpreted their own social reality in the context of their shared family realities. Hence, meaning was created through their interaction. We did not search for one objective truth within the families but rather perceived all views and experiences expressed by children and their parents as equally valid, even if they appeared contradictory [18]. The interview was initiated by the following introductory question:

“I would like to ask you [name of child] if you remember the time when you were diagnosed with diabetes? Will you tell me about it?” The child was encouraged to tell his/her story in their own words and with a focus on whatever they perceived important. If the child did not remember fragments of the story, or did not remember the time of diagnosis at all, the parents were asked to provide supplementary information. To gain insight into details and broader perspectives of the story, follow up questions such as “can you tell me more about…?” and “What made you think that…?” were asked continually.

To continue to provide a child-friendly frame, a significant amount of time was spent on two dialogue exercises (described in detail below). The exercises were designed to facilitate discussions within the family at a level appropriate for the age of the child. Following the dialogue exercises, six supplementary questions were posed to the family. Although we expected that most answers to the supplementary questions would be revealed during the exercises, we wanted to ensure that these questions had been addressed across all interviews. The first two questions focused on the optimal outpatient visit: “Can you tell me about the best outpatient visit you experienced?” and “What made it good?” The next two questions focused on the less optimal outpatient visit: “Have you ever felt that an outpatient visit wasn’t a good experience?” and “What happened?” The last two questions focused on implications for practice by inviting the family to share their ideas for future benefit of other families with childhood diabetes: “Is there anything you find important to pass on from your present clinic to the new SDCC pediatric clinic?” and “If you could suggest something for the pediatric clinic, what would it be?”

During the interview, additional probe questions were used to promote a dialogical interactive process between the family members. Probes such as: “Is your experience similar to your mother’s?”, and “You look like you disagree. Do you?”, were used to elicit responses from all family members without asking questions in turns.

#### Dialogue Exercises

**Figure 1:**
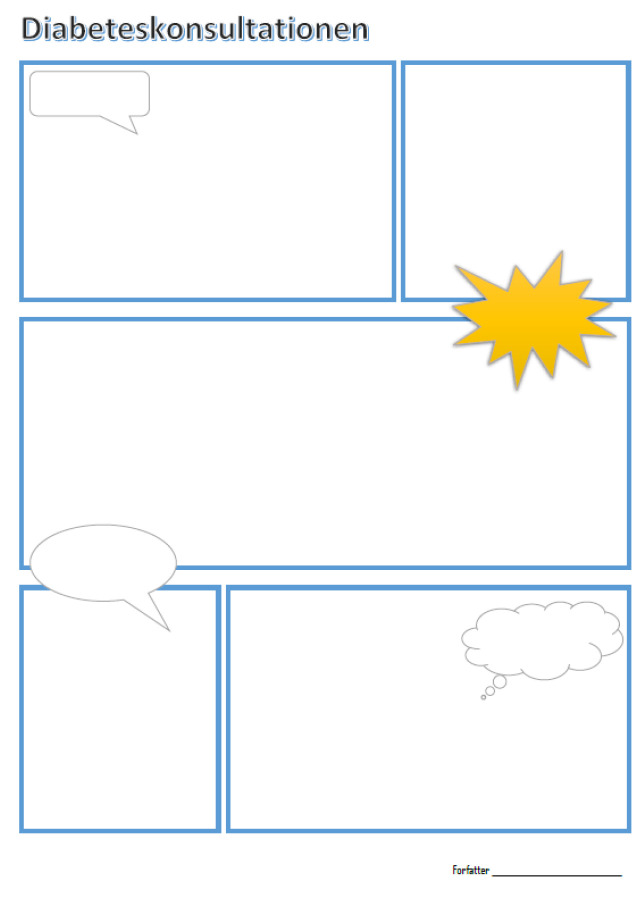
Template for drawing exercise.

For the drawing exercise, each family member was asked to fill in a blank cartoon (Figure 1) about their experience with their regular outpatient visits. Subsequently, family members were asked to share the thoughts underlying the drawings, the goal being to stimulate discussion between the family members by focusing on the similarities and differences in their individual experiences.

Following the drawing exercise, we conducted a preference ranking exercise inspired by the participative ranking method [19]. The intention was to challenge the perceptions of their individual priorities regarding diabetes treatment and to use differences and similarities to facilitate discussion.

Each family member received an envelope containing eight pieces of paper each with one statement and a small drawing to help the young children remember the content. All statements were read aloud by the interviewer. Family members were then asked to rank the statements by arranging them with the most important statement on top. Subsequently, the family members were asked to agree on a common list to encourage articulation of the rationales behind their individual rankings. Before introducing the exercises, the participants were informed that their thoughts, experiences, and considerations were essential and that the drawing and the ranking order were less central to us. The ranking statements were inspired by topics from a synthesis of qualitative studies on children’s, young people’s, and parents’ priorities for care [3]:

1. The health professionals ask questions about my diabetes;
2. The health care professionals ask questions about my school/daycare;
3. I know the health care professionals;
4. The health care professionals seem interested in how I feel;
5. I have influence on what to talk about;
6. I have influence on my treatment goals;
7. I can get in contact with the health care professionals when I need to;
8. My teachers are taught how to help me.

For parents, “my”, “me”, and “I” were replaced with “my child” or “my child’s”, but the wording was unchanged to allow children and parents to discuss on the same basis.

All family interviews were audio recorded and transcribed verbatim by the first author and two student assistants. Prior to the interviews, written informed consent was obtained from all participating parents on behalf of themselves and their children. Furthermore, they agreed that their drawings from the drawing exercise could be shared. According to Danish National Ethical Committees, the present study did not require ethical approval. This study complies with the ethical guidelines of the Declaration of Helsinki [20] and was approved by the Danish Regional Data Protection Agency. Registration number: P-2019-660.

### 2.3. Analysis

Systematic text condensation, as described by Malterud (2012), was used to analyze the data stepwise [21]. The different analytical steps of the systematic text condensation were performed repeatedly during the analysis. This led to several changes and modifications before agreement on the analytical themes occurred. Initially, in the first step, all 13 audio files were listened to, and the transcripts were read by two experienced qualitative researchers, who were not part of a clinical team (LNJ and DG), to gain overall familiarity with the data. Preliminary thoughts on the data were thoroughly discussed among the authors. Two main themes—communication and social relations—were identified as recurring across the data and were used as foci and coding frames for subsequent analysis. In the second step, all transcripts were re-read, and meaning units connected to communication and social relations were identified by LNJ. In the third step, to ensure that all data informing the two main themes had been included, DG read the meaning units to validate the analysis and to check them against the transcripts. The meaning units were then condensed in collaboration between LNJ and DG, and while carefully preserving the essence of each meaning unit, each was labeled with a code. In the final step, data were reorganized according to the codes and categorized into distinct analytic themes which were checked against the transcripts and discussed among the authors.

The first author identified illustrative citations in the transcripts and decided, in collaboration with the other authors, on those representing the themes in the best way.

No new overarching themes appeared during the last two interviews, at which time the researchers agreed that data saturation had been reached.

## 3. Results

Following coding and analysis of the data, with a specific focus on communication and social relations, nine analytical themes were identified. Table 2 provides an overview of the nine themes identified in the present study. Each theme is elaborated below.

### 3.1. Discrepancies in Perception of Diabetes Tasks

The families had the common experience of the health care professionals sometimes forgetting that clinical practice may be unfamiliar to the families. The complex knowledge and required actions that seem natural to health professionals who work with diabetes every day are not necessarily obvious to the families. Because of this, it can be difficult for the families to keep up—regarding both information delivery and concrete actions. One mother reported finding it difficult to understand the calculations explained in the consultations:

*“And that is where I opt out… When things go too fast sometimes, and they are so into all that (…) ‘…and then you take her numbers and her weight and multiply and divide, and then you get the number 10’. And I’m just thinking ’What are you talking about?!’”*

(Mother of a 9-year-old)

In terms of concrete actions, finger-pricking and blood samples were a source of worry and discomfort for the parents, especially parents of younger children. They unanimously reported a feeling of violating their children when they had to restrain them to perform the required tests:

*”… Obviously, it’s a lot about these pricks (…) they’re a strong presence… And that you have to hold on to him gives you a feeling of assaulting your child…”*

(Father of a 4-year-old)

One mother reported feeling overwhelmed when her son was put on the continuous glucose monitoring system. Although this is everyday life when working with children with diabetes, it can feel like a devastating and pivotal event for families newly introduced to their child’s diabetes diagnosis:

*… He got the Libre right away… but that was also quite overwhelming, because you don’t know anything, and then you get the message about him having diabetes, and they whip that thing onto his arm! ‘Oh! Ok! So he’ll wear this for the rest of his life?’…*

(Mother of a 4-year-old)

The children often experience that the obvious is not necessarily obvious to everyone. One young boy described how he, on the third day of hospitalization, had asked why he would have to do certain tasks and was given the answer: “Because you have diabetes.” Only then did he learn about, or perhaps grasp, his diagnosis: “*… Because I have diabetes?… I was like; ‘What?’”* (9-year-old)

The fact that receiving new complex information about diabetes can be difficult to process was also a recurring topic among the parents. They talked about having been flooded with information shortly after learning that their child had diabetes and not being able to comprehend it. This was especially related to information about diet, which some of the families would have preferred to have received at a later time.

*“I don’t remember what the dietician said on the second day. Like really, I don’t remember it! While we were hospitalized, we were flooded [with information]. And then after we had been discharged, it felt as if we were on our own.”*

(Mother of a 9-year-old)

### 3.2. Talking about Adult Things, but the Children Listen

Children as well as parents addressed issues related to what was articulated as “adult talk”. Adult talk was described as a dialogue between parents and health professionals that did not include the child, either because the child did not understand the content or because the conversation addressed issues (e.g., technical advice on a pump) that the child did not need to be involved in. In both cases, the children experienced the adult talk as predominant and boring. The parents were aware of their children’s experience and reported that, when the children were bored, they tended to become restless and impatient, making it difficult for the parents to concentrate on the conversation. To ease the children’s boredom and the parents’ lacking concentration, the parents would have liked to have had the option of letting their children leave the room to play—ideally in a staffed playroom, but a table with interesting toys would also have worked.

*“… so for those [children] who didn’t want to sit and listen to what we were talking about, it would have been quite nice, if there had been something to keep them busy.”*

(Mother of a 12-year-old)

Although most children experienced this type of conversation as boring and undesirable, parents expressed the need to have the possibility to discuss technical issues and to clarify any questions they might have.

*“… There are so many things we have to talk about that a 5-year-old doesn’t understand or have an interest in. I mean, he doesn’t care about numbers, but it’s his body, which makes him the only one able to feel and experience it [diabetes]”*

(Father of a 5-year-old)

For parents and health professionals, it is vital to be aware that the children are listening to the conversation—even when they appear to be occupied with, e.g., drawing or doing a puzzle. The following excerpt from an interview with an 8-year-old boy illustrates a potential consequence of listening, but not quite understanding:

*Sometimes, it would be nice if I understood some of the things. I mean, if I was included in the conversation.” Interviewer: “So you hear words that you don’t understand—is that right?” “I hear words I know; but they are arranged in an order I don’t understand*

(8-year-old with diabetes)

### 3.3. The Importance of Spoken and Written Words

The possibility for parents to easily access their child’s medical records has led to the records no longer being solely a professional tool for the health professionals, but also a source of information for the families. Thus, wording and descriptions included in medical records should be considered carefully and with their potential impact on families in mind. One mother explained how she and her husband were offended by reading in the medical records that the doctor doubted that the parents had understood what was said:

*We were upset about what had been written in the medical records. That the doctor doubted that we understood what had been said… and I think that we were both quite offended.*

(Mother of a 5-year-old)

Such inconsistencies between the parents’ experience and what they subsequently could read in their child’s medical records were reported by a few families. Difficulties in relating to descriptions of their child’s behavior during the consultation caused reluctance about bringing their child to future consultations, due to concerns about how the child would be viewed, interpreted, and described in the medical record:

*Once he [her son] was angry. And when we came home, I could see in the medical record, that the doctor had written this and that: it was quite a label he had gotten—‘that he was a hothead’ where I was like; ‘ok, it’s just a snapshot.’ Then I don’t feel like bringing him for the next visit.*

(Mother of a 4-year-old)

Several parents, especially mothers, reported that their child’s HbA1c was highly important to them. They stressed that the aim of obtaining low HbA1c measures was not driven by health professionals but was solely a manifestation of their own expectations of successful diabetes management:

*… So she had her long-term blood sugar measured, and that’s probably what worries me the most. You can’t have perfect blood sugar, that’s impossible, but we are aiming for her not to have too high blood sugar, right? It’s just your own ambitions… or imaginations about what you want the numbers to be, right?*

(Mother of a 10-year-old)

Because of the perceived significance of the HbA1c measures, mothers explained how the HbA1c results could feel like a general assessment of their parental skills. They explained that the way the results are communicated can make a difference in their perception of how they manage taking care of a child with diabetes:

*… Of course, they have to tell you, if the HbA1c all of the sudden is very high and something has to be done about it. But it’s important that it’s said in a good way, so it doesn’t feel like an examination.*

(Mother of a 5-year-old)

### 3.4. Confusion about Division of Responsibilities

The families reported feeling that they were being held responsible for setting the agenda for the consultations. Still, most families agreed that it was not important to them to decide the topics to be discussed in the consultation. Quite the contrary, the families wished that the health professionals would bring up different topics relevant to discuss with their family. The families reported not being familiar with the cause of diabetes, and as they explained it:

*There are a lot of things we don’t know, and if we don’t know we don’t know it, then we’re unable to ask about it. Maybe during the first year, you could say; ‘we need to make sure to have touched upon these themes’ so that you know that there’s some sort of structure to it.*

(Mother of a 9-year-old)

Navigating in both the health system and the social system, the families felt burdened by the responsibility of having to keep track of paragraphs, prescriptions, and public subsidies. One mother of a 9-year-old girl reported being at the pharmacy to pick up her daughter’s insulin, when she was told that there was no prescription for the medication. She had not been informed that the prescription had to be renewed every two years. Additionally, she explained how ordering the medical aids was tiresome and time-consuming:

*Now we have to send an e-mail to the hospital, and they order it at the pharmaceutical company and then they ship it to our home (…). And then we have the i-port and the whole follow-up stuff, which we order at [name of company], and they have the pump. And then, if there are grants from the municipality, we have to order bandages, and everything else from a third place, so I think it’s quite confusing.*

(Mother of a 9-year-old)

Further, the parents felt they had been assigned heavy responsibilities, in that they had to convey complex information from the hospital about their child’s diabetes to their daycare or school. They experienced complex challenges in this intersection between the two different systems—the health system and the social system—and felt they had been placed in the middle and held responsible for making both ends meet.

[About having to inform the kindergarten] *… there’s so much to figure out at the beginning… and at the same time we must apply at the municipality. And what do you do with work and all that?… so in one way or another, it would be nice to have some guidance in what to do and everything you have to think of.*

(Mother of a 5-year-old)

Many parents reported that the courses held to inform caretakers and teachers had not had the desired effect due to lack of participation on the part of the intended target groups. Furthermore, the knowledge offered during the course was only acquired by a few persons, and frequent changes in daycare and school staff have meant that this important information can easily vanish because it is not embedded at the organizational level.

*(…) then only two persons attended the courses and in the end it will be us [parents] who teach the caretakers. Many of the new caretakers were taught by someone else who was insecure, and we’ve had periods where we were told, that we couldn’t send him to day care because there weren’t any that day to… [take care of him].*

(Father of a 3-year-old)

For such complex matters, the families stressed the need for a coordinator, who could relieve the parents as well as the doctors. The doctors can manage clinical matters, but others can talk to the families about non-clinical issues such as friends, family life, and legislation and, in this way, *“… let the doctors be more doctors.”* (Father of a 3-year-old)

The parents additionally stressed the need for stronger coherence across sectors and a more coordinated and coherent process that is independent of the parents and their resources.

*… whether it is the local municipality or the Region that has to say ‘when a child gets diabetes, the leader and the two primary care takers have to get a course,’ because there is some basic knowledge you need to know, and you have to receive that knowledge from professionals. The parents cannot provide it … (…) such a collaboration is wishful thinking.*

(Father of a 5-year-old)

Some families reported that other families had been offered different types of support than they had been offered. This was to be expected when the families belonged to different hospitals, but more difficult for the families to accept was when families belonging to the same hospital were offered different support.

*They [another family] had their nurse explaining about diabetes in the day care… And so we were kind of left with a ‘so is that really depending on which nurse you have?’ Because we have a different nurse.*

(Father of a 5-year-old)

### 3.5. Relief When Someone Eases the Burden

Non-clinical aspects of childhood diabetes were generally perceived to be a significant burden to the families. Especially at the time of diagnosis, the families shared the experience of the biological aspect being well taken care of, but the psychological and social aspects—adapting to the new situation, applications, informing the school, etc.—being left to the families to deal with on their own. The families described situations in which someone had relieved them of this burden by offering to inform the school or fill in an application form. Such tangible acts may appear insignificant, but the feeling of being relieved had tremendous value for the families, and words such as “magic” and “gold” were used to describe these actions:

*I can’t remember her name, but she was worth her weight in gold! (…) It was all magical. It was fantastic to have things done for you.*

(Mother of a 9-year-old)

This quote illustrates how relatively small gestures can have a major impact on families’ overall experience with the pediatric clinic.

### 3.6. Courtesy When Visiting the Clinic

The families generally reported positive relations with the health professionals. The positive relations were manifested by families always feeling welcome when visiting the pediatric clinic. They felt secure and that they were in competent hands. Some parents described how going to see the nurse was actually enjoyable:

*There was just such a warm welcome every time, that was great, because it eliminates the hospital atmosphere. The fact that it is actually quite nice to come to see her [the nurse], right?*

(Father of an 8-year-old)

The fact that the children were recognized and received small gifts further added to the feeling of being welcome. The parents reported that the children feel good about visiting the clinic, and some parents said that their child always looks forward to seeing the nurses and receiving a small gift. The importance of this positive relation was stressed and considered vital in helping the children feel secure in the clinical setting:

*You feel really well treated, I think. They are very, very nice and welcoming. And that’s really important as we deal with our kids.*

(Mother of a 3-year-old)

### 3.7. Understanding of the Family Context

Even though the child has a chronic illness requiring a vast amount of attention, family life still comprises busy schedules with jobs, school, leisure activities, school meetings, birthday parties, etc. The parents emphasized that the children’s diabetes is part of the family context, and because it is a permanent brick in the large, complex family life puzzle, treatment must be tailored to fit the individual needs of each family. If medical standards and advice are not adapted to the actual circumstances within each family, chances are that they will not be sufficiently implemented and thus will not have the desired treatment effect. The families felt the health professionals made an effort to accommodate the need for customized treatment:

*But then they are responsive to the fact, that it just won’t work in our everyday life… because with the other sensor, you always have to look at the pump to see his blood sugar, and that’s just annoying, so you end up disturbing him even more.*

(Mother of a 4-year-old)

During visits to the pediatric clinic, the parents emphasized the need to have plenty of time. One mother of a 3-year-old reported: *The more you try to hurry them [the children], the worse it gets.*

Having enough time was perceived as important to making the children comfortable and establishing a personal relation between the family and the health professionals. The families appreciated that the visits were not rushed and experienced the health professionals as patient and attentive:

*… they are really good at not rushing, I mean, you always feel there’s enough time available! And when they talk with [name of child], they are SO patient; you know; they listen—they want to talk and listen and wait and listen.*

(Mother of an 8-year-old)

### 3.8. Importance of Continuous Personal Relations

Throughout the interviews, families underlined the importance of seeing the same few health care professionals for their regular outpatient visits. For children and parents, the personal relation provided a feeling of psychological safety, which, in turn, laid the foundation for establishing trust between the family and the health professionals. This mutual trust was considered crucial when addressing personal and difficult aspects of the diabetes treatment. The mother of a 10-year-old explained:

*… it’s also about establishing a relation or trust as a kid. Trust in the adult, right? When you sit and talk about something so personal and also difficult, then this is important.*

(Mother of a 10-year-old)

Her view was supported by the parents of a 3-year-old. They stressed that, especially for the children, knowing the health care professionals is of the utmost importance:

*… it is important that you see the same doctor and the same nurse. It’s safe especially for the kids (Father of a 3-year-old). …Now he’s familiar with them and knows what will happen, so it’s very important, that it’s someone he knows.*

(Mother of a 3-year-old)

One family was about to move, and their willingness to spend increased travel time to visit their usual health professionals revealed just how important this relation is to the families:

*We will be moving in a couple months and have been talking about being willing to drive the extra distance to get to the [hospital], to get to see the ones you know and stick with the place where we started.*

(Mother of a 4-year-old)

Furthermore, the feeling of continuity was enhanced by the recurring personal relations. Some families were worried that the personal relations with the health professionals would be compromised in the new large clinic. The families reported no significant difference in communication with the nurses and doctors. The children valued being able to continue the communication from their last visit and that they did not have to explain the same things to different health professionals.

*Just so that you don’t have to talk to different people. I know that the ones I have been talking to, more or less know everything I’ve been telling them, so that I don’t have to always explain it again and again.*

(10-year-old)

One father suggested the possibility of occasionally consulting health professionals other than those they normally saw for consultations. He thought it could be useful to get different perspectives on, e.g., new treatment options.

*… it might be quite good to talk to different doctors. I actually think you get different points of view and maybe different dialogues about it; it can be technical, it can be about how things function… the opportunities available or the like.*

(Father of a 4-year-old)

### 3.9. Need for a Facilitated Peer Network

Most parents addressed the need to meet up with other families with a child with diabetes. They stressed that the network meetings should be facilitated, and that participation should be voluntary. There were no clear wish for the format of such meetings, but a common wish was that the pediatric clinic would take the initiative. Although there was no expectation that the health professionals should facilitate all network meetings, it was suggested that they regularly attend the meetings to: *Tell us something about; innovation and news.* (Father of a 10-year-old)

As an alternative to planned, facilitated network meetings, one father suggested that the pediatric clinic could facilitate contact with other children with diabetes of the same age living in the same neighborhood, thus enabling them to form informal networks with other families. For now, this need is being met in social media groups, where the large number of members ensures that someone will most likely have had experiences with the same issues:

*… when you ask in a [Facebook] group with 500 members, there is definitely someone who has gone through the same situation and can say ‘well, it’s all normal; it happens to us too’…*

(Mother of a 4-year-old)

However, the parents were aware of the missing professional input and would like to be able to discuss the issues with someone with the right expertise:

*It’s just really nice when it comes from someone who actually knows something about it.*

(Father of a 5-year-old)

The families were aware of the different events offered by patient organizations but made it clear that they preferred that all diabetes-related events originate from the pediatric clinic:

*… Meeting other families.. I actually think that’s what the Diabetes Association arranges… But I think, it suddenly strikes me, that we’re attached to the outpatient clinic more and more… Because we know them and they’ve been there throughout.*

(Mother of a 9-year-old)

## 4. Discussion

In the present study, we explored what children with diabetes and their parents perceived to be important in relation to their outpatient visits at a pediatric diabetes clinic. Five aspects elaborating on the importance of communication were identified: 1. Discrepancies in perception of diabetes tasks, 2. Talking about adult things, but the children listen, 3. The importance of spoken and written words, 4. Confusion about division of responsibilities, and 5. Relief when someone eases the burden. Additionally, four aspects expressing how the importance of social relations was manifested in the pediatric diabetes clinic were identified: 6. Courtesy when visiting the clinic, 7. Understanding of the family context, 8. Importance of continuous personal relations, 9. Need for a facilitated peer network.

Communication skills and communication style have previously been found to have a major impact on how clinical visits are experienced [8]. A study by Lowes et al. (2015) explored children’s, adolescents’, and carers’ perceptions of visiting a diabetes clinic and found that good communication skills had a major impact on the clinical experience [8]. Other studies have shown that communication styles vary among health professionals [6,22,23]. In our study, no significant differences were identified between nurses and doctors. Previous research has described how parents feel as if the health professional is talking to a colleague rather than trying to use terms that the family can understand [22]. The results of the present study support this finding by revealing how the children and their families felt that the clinic was a place where everything was familiar to the health professionals, but that the health professionals would often forget that the families were new to these different practices as well as to the clinical language and, hence, required thorough guidance and explanation. A study from 2014 investigating the challenges of optimizing glycemic control in children with type 1 diabetes found that parents and children often experience anxiety before going to the clinic to receive the HbA1c results [24]. Our results do not support this finding, although especially the mothers in our study reported feeling evaluated by the measures and that it was of the utmost importance that the numbers be presented “in a sensible way.” In the present study, health professionals choosing appropriate words was highlighted, as both the children and their parents felt that “difficult words” or complex calculations, which were sometimes used during consultations, made it difficult for the children to engage in the conversation. We found that inclusion of the children was important to the families. However, the parents simultaneously expressed their need to discuss issues related to their child’s diabetes without disturbance from the child. The identified challenge of receiving new and complex information while having to attend to their, perhaps impatient, child supports previous research which suggests that this can imply that parents leave the consultations with unanswered questions [22]. Coordination of diabetes care has been found to be of great value to parents of children with diabetes [3]. Our identification of confusion about the division of responsibilities during consultations and between systems confirms the parents’ need for coordination and stresses how much they appreciate it when someone eases their burden in relation to applications, knowledge division, and adapting to having a child with diabetes. No distinct patterns related to age, time since diagnosis, or the two different clinical settings were identified. However, there was a tendency for participants belonging to the smaller outpatient clinic to emphasize that experiencing continuity and having a personal relation with their health care professional were fundamental to their clinical visits. The finding that continuity with doctors and nurses is crucial supports results from other studies showing that lack of continuity at clinical visits makes it difficult for children and adolescents to build an ongoing relationship of trust with health care professionals [3,25]. It is worth considering whether personal relations will be more difficult to establish in the larger SDCC clinic. The reported desire for continuity may be somewhat contradictory to wanting the opportunity to get a second opinion. However, the opportunity to see other health care professionals and perhaps get a second opinion could be combined with the possibility of networking with other families in, e.g., group consultations [26]. In accordance with existing knowledge, the children in the present study expressed no particular desire to join peer networks. This may be because they do not want additional focus to be placed on diabetes [3]. The perceived importance of courtesy when visiting the pediatric clinic has not previously been addressed explicitly in the literature. However, the significance of delivering, e.g., HbA1c results in a sensible way [22] and clinicians’ style of interaction [3] is closely related to personal relations, mutual respect, and how the health professionals generally approach the families. Courtesy can also be expressed in how well health care professionals manage to adapt the treatment to fit the needs of the individual families. In accordance with the existing literature, such flexible treatment adapted to the family situation has been shown to be important to families [3,24,25]. Furthermore, a study of adolescents suggested that, if health professionals provide advice that does not fit the everyday life of the families, this advice is likely to be ignored [25]. Obtaining a balance between optimal medical treatment and individual family preferences requires that the health professionals and families share responsibility. The families have the responsibility to clearly articulate their wishes and needs, while the responsibility of health professionals is to be responsive to the current circumstances within each family.

### 4.1. Methodological Considerations

Inevitably, there are some limitations associated with the method of data collection. However, the present study adds to the existing body of knowledge by offering profound insights into the families’ experiences, needs, and preferences in relation to the pediatric diabetes clinic. The choice of asking nurses to recruit families was an essential, and ethical, part of our research process. The families were approached by a familiar and trusted person and only contacted by the researcher when they had already agreed to participate. However, we cannot rule out a potential recruitment bias if the recruiting nurses, consciously or subconsciously, recruited families who appeared to have a surplus of energy, would feel comfortable with a researcher visiting them in their home, and were somewhat satisfied with their clinical visits. Further, we did not obtain data on socioeconomic status and clinical parameters, and thus we cannot elaborate on whether the family’s experience with the clinic seems to be associated with the child’s HbA1c or glucose monitoring approach. Although these data were not obtained, our impression is that the sample in the current study mainly consists of well-regulated children and well-educated, working parents with a, primarily, Danish background. This means that our findings may not be representative of the experiences of all Danish families with pediatric diabetes and may not apply to other cultural contexts. In particular, the experiences of families with cultural backgrounds other than Danish, families with fewer socioeconomic resources, and families that are not satisfied with the clinical visits should be explored further.

Traditionally, research has suggested that children six years and under may find interview situations difficult and provide only yes or no answers [27]. However, more recent research shows that preschool children hold their own views and opinions and are capable of expressing valuable perspectives regarding their contexts and worldviews [27]. In the present study, we considered children to be capable and valuable experts on their own lives and, thus, invited all children to participate in the conversation and exercises according to their own inclination and at their own pace. Many of the children used the possibility of temporarily withdrawing from the interview to take a break. For instance, a very young child went to his room to play and returned after a short while with a book he wanted the interviewer to read to him; a teenager went out into the garden and played with a ball, but he also returned to the interview after a short while. Although children are considered capable, it is important to recognize that they may be more vulnerable than other participants to unequal power relations with an adult researcher [28]. Therefore, it was our priority to meet the children and their families at home where they felt secure about sharing their experiences. Interviews conducted at the hospital have been shown to be somewhat biased in favor of the hospital and the staff [29]. The fact that the family was the host and the interviewer was the guest initially evened out some of the power imbalance. As a further attempt to reduce any power imbalance, all interviews were initiated with an introductory question about the time when the child was diagnosed. The time of diagnosis is an important and epoch-making event for the families, and their experiences from that period are often imprinted on their mind [30]. Because the interviewer knew nothing about the time when the child was diagnosed, the interviewer was left to play a more passive role at the beginning of the interview while the families did the talking.

Some of our findings might not have been revealed if other less flexible and less comprehensive data collection methods had been used. For example, interviewing the children and parents separately would not have shed light on reflections on the discrepancies between the child and the parents, which were revealed when, e.g., the parents provided an answer that the child then objected to. During data collection, it became clear that using family interviews meant that the parents interviewed their child and vice versa. This is a known advantage of family interviews [18]. In quite a few interviews, the family members spontaneously began interviewing each other. This happened especially in situations where their perceptions differed, and they therefore needed an elaboration of the other’s point of view. One example of such dynamics was when a young girl disagreed with her mother’s priorities and challenged them. Another advantage of the family interview was observed when the parents’ narrative about the time of diagnosis was often addressed directly to the child, and some of the older children diagnosed a few years back may have heard their parents tell this story in its entirety for the first time. Some parents were surprised that their child did not remember the time, and this introductory question about the time of diagnosis therefore appeared to enhance the family experience by creating a common understanding of what happened when the child received the diagnosis. The aim of the dialogue exercises was not to analyze the drawings and rankings or to consider them as true descriptions of experiences, but rather to encourage and facilitate conversation between the child, the parents, and the researcher. Although we found the dialogue exercises to be useful as a means of involving the children in the conversation, the young children’s voices were not sufficiently evident in the present study. Subsequent individual interviews with the children could have resulted in other details or perspectives being added to the data.

### 4.2. Implications

Our findings provide valuable information for directing attention to the underlying experiences and perceptions of children with diabetes and their parents. Based on the families’ experiences, to promote optimal experiences of pediatric outpatient visits, we outline the following:

Some of the focus points (left and middle columns in Table 3) have the potential to make an immediate difference in the clinic without introducing comprehensive interventions or imposing tough financial priorities. By bringing awareness to the importance of, e.g., courtesy and clear communication about responsibilities, the health care professional can keep in mind how important this is to the families. Introducing days on which families with children of a specific age are scheduled for clinical visits may require some logistical changes. Such changes would allow families to meet other families in the waiting area, as a way of initiating informal networks for families with children of the same age with diabetes.

Other findings may not be directly applicable in a clinical setting where resources are sparse. For example, employing persons to ease the non-clinical burden would imply that other initiatives would have to be given a lower priority. Such dilemmas were expected, as we did not ask the families to relate to economic or personnel resources, but simply to relate to their personal needs. As regards promoting optimal communication, it is important to recognize that although health professionals can regularly make sure that families, including the children, understand what is going on, the families are responsible for communicating clearly when there are things they do not understand or things they are not confident about. In conclusion, the present findings encourage reflection on how to improve communication and underline the importance of establishing a continuous and personal relation between families and health care professionals to improve families´ experience with pediatric outpatient visits.

## Figures and Tables

**Table 1:** Participant characteristics.

Family	Child Gender and Age	Participating Parent(s)	Duration of Diabetes (Years)	Hospital
1	F10	mother	4	A
2	F15	mother	9	A
3	M8	mother and father	7	A
4	F10	mother and father	6	A
5	M3	mother and father	1.5	A
6	M12	mother and father	0.5	A
7	F9	mother and father	3	A
8	F10	mother and father	1	B
9	M4	mother and father	2	B
10	M5	mother and father	0.5	B
11	M4	mother	2	B
12	M9	mother	1	B
13	F8	mother	7	B

**Table 2:** Analytical themes identified across family interviews.

Communication	Social Relations
1. Discrepancies in perception of diabetes tasks	6. Courtesy when visiting the clinic
2. Talking about adult things, but the children listen	7. Understanding of the family context
3. The importance of spoken and written words	8. Importance of continuous personal relations
4. Confusion about division of responsibilities	9. Need for a facilitated peer network
5. Relief when someone eases the burden	

**Table 3:** Focus points for promoting optimal pediatric outpatient visits.

Be Aware That	Do	Aim for
Clinical procedures and information may be unfamiliar to the families	Approach the families with courtesy	A continuous personal relationship between health professionals and families
The children listen, even when you are talking about “adult things”	Adapt the treatment to fit individual family needs	Easing the burden of, e.g., administrative tasks
The notes in the medical records are available to the families	Clearly define which responsibilities belong to the parents, health professionals, or others (e.g., social worker)	Generating opportunities for facilitated peer networks
	Have a structured agenda (based on, e.g., age, blood glucose level, and time since diagnosis) to ensure relevant conversations	

## Data Availability

Data are available from the authors on request.
